# Using a birth cohort to study ageing: representativeness and response rates in the National Survey of Health and Development

**DOI:** 10.1007/s10433-013-0258-8

**Published:** 2013-01-22

**Authors:** M. Stafford, S. Black, I. Shah, R. Hardy, M. Pierce, M. Richards, A. Wong, D. Kuh

**Affiliations:** MRC Unit for Lifelong Health and Ageing, 33 Bedford Place, London, WC1B 5JU UK

**Keywords:** Attrition, Non-response, Education, Cognition, Home visit

## Abstract

Britain’s oldest birth cohort study, the MRC National Survey of Health and Development (NSHD) provides data to explore life time influences on ageing. The latest data collection was undertaken between 2006 and 2011 when study members were aged 60–64 and consisted of postal and pre-assessment questionnaires to eligible study members, followed by invitation to attend one of six clinical research facilities (CRFs) across the UK for clinical assessments, and dietary diaries and activity monitors in the days following the CRF visit. The option of a home visit for clinical assessments was provided if the study member refused or was unable to attend the CRF. We examined response and attrition, here describing rates overall and for postal and clinical assessment modes of data collection, identifying socioeconomic and health-related predictors of response, and assessing the continued representativeness of the sample. In total, 2,661 (84 % of the target sample) responded. Lower educational attainment, lower childhood cognition and lifelong smoking independently predicted lower likelihood of both overall response and CRF cooperation. At 53 years, not owning one’s home and not being married predicted lower likelihood of overall response whereas manual social class and obesity predicted lower likelihood of CRF cooperation. Providing for collection of biomedical data in the home and use of assessment instruments and modes to retain study members with lower education attainment, lower cognition and poorer health behaviours should be priorities for helping reduce attrition amongst vulnerable ageing study members.

## Introduction

Understanding ageing trajectories is a research priority requiring longitudinal data, preferably from the whole of life (Medical Research Council 2009). Early life, childhood circumstances are associated with physical, cognitive and social functioning and chronic disease risk in later life (Barker 1999; Ben-Shlomo and Kuh 2002). Birth cohort studies enable prospective description of life time exposures and their impact on changes in health in later life. The robustness of the evidence they contribute depends on their continued representativeness of the target population and freedom from bias due to avoidable attrition. Achieving high response rates among both young and older participants is becoming more of a challenge for survey investigators (Porter 2004).

The oldest British birth cohort, the MRC National Survey of Health and Development (NSHD), recently celebrated its 65th birthday and completion of its 23rd follow-up (Kuh et al. 2011). The focus of the most recent data collection (at ages 60–64) focused on changes in physical and cognitive function and mental health, biological samples (blood, urine and saliva) for biomarkers of ageing, and the first ever imaging of participants to assess bone and body composition, and cardiac and vascular structure and function. These clinical assessments required a visit to a clinical research facility (CRF) although a more limited set of measures was taken at home if preferred. A postal questionnaire preceded the visit. This data collection was far more extensive than previous follow-ups which had only involved either a home visit from a research nurse or a postal questionnaire and had generally achieved response rates of over 80 % (Wadsworth et al. 2006).

There was concern that this more intensive data collection would affect the overall response rate, either decreasing it because the clinical assessments would put some study members off or increasing it because other study members would value the chance of a more comprehensive biomedical examination and feedback than had been previously offered, as found in another study (Mein et al. 2012). Analysis of the feasibility phase of the latest NSHD data collection provided weak evidence that those who had an existing doctor diagnosis or had undergone hospital procedures related to coronary heart disease were more likely to participate in the clinical assessment (Kuh et al. 2011). This raised the question of whether the socio-demographic and health predictors of participation in the clinical assessment differed from those of participation in other elements of the study.

Many studies of ageing start in adulthood, have baseline non-response, and are unable to investigate earlier predictors of attrition as the cohort ages. The few long-term ageing studies that have examined attrition did not find consistent predictors, although there is a general trend towards greater attrition amongst those in poorer socioeconomic circumstances and poorer health (Slymen et al. 1996; Young et al. 2006). A systematic review of attrition in longitudinal studies of the over 65 s (Chatfield et al. 2005) revealed that cognitive impairment and advancing age were the only variables that independently predicted attrition. Lower cognitive scores in childhood have also been related to non-response to a postal questionnaire around 40 years later in the Children of the 1950s cohort set in Aberdeen (Nishiwaki et al. 2005). Previous bivariate analysis of attrition in the NSHD indicates that factors in both childhood (including male gender, household crowding, low father’s social class, lower cognitive test scores and teacher assessed anti-social behaviour) and adulthood (including low educational attainment, low social class, living in rented accommodation, work stress and not belonging to clubs or associations) are associated with avoidable non-response in adulthood (Wadsworth et al. 1992; 2003; 2006). Although several ageing studies use a variety of assessment modes including clinical assessments in the home or research clinic, face-to-face interviews and postal questionnaires, differences in the response rate for each mode by socioeconomic and health characteristics have received little attention.

Thus, few ageing studies have examined independent lifetime predictors of attrition, or of participation in distinct assessment modes. The aims of our paper are to (i) describe response overall and for different components of the data collection at age 60–64 years; (ii) examine the extent to which response was differentially associated with (a) socio-demographic factors in childhood and young adulthood, (b) socio-demographic factors, health and health-related behaviours from early adulthood to midlife; and (iii) compare the socio-demographic and health characteristics of NSHD participants at age 60–64 years with the UK born population of the same age.

## Methods

The NSHD is based on a social class stratified sample of 5,362 births of all singleton births that occurred within marriage in a week in March 1946 in England, Scotland and Wales. Previous follow-ups occurred approximately every 2 years in childhood, and the previous main data collections in adult life were at 26, 36, 43 and 53 years (Wadsworth et al. 2006).

The 60–64 year data collection consisted of a postal questionnaire to eligible study members, followed up (between 2 months and 2 years later) by invitation to attend one of six CRFs across the UK for assessments or, if they were unable or unwilling to travel, to have a research nurse visit the study member at home (Kuh et al. 2011). A reduced set of clinical measures was carried out for those visited at home. All participants were also asked to complete a pre-assessment questionnaire before the visit.

Ethical approval for the study was obtained from the Greater Manchester Local Research Ethics Committee and the Scotland A Research Ethics Committee. Written, informed consent was obtained from the study member for each component of data collection.

### Defining response and cooperation rates

**Fig. 1:**
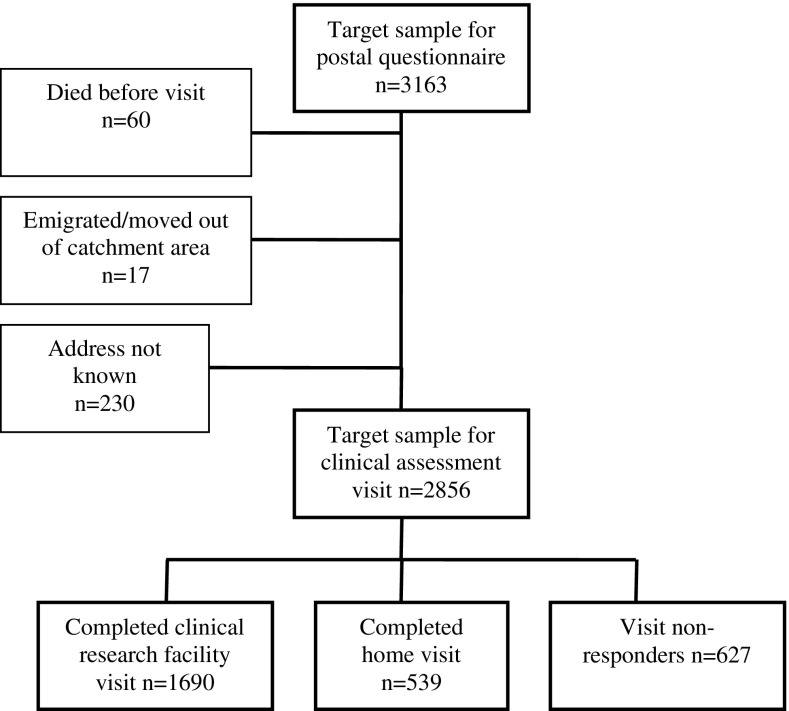
*Flow chart* summarising response status at 60–64 years

Study members were defined as eligible for the 60–64 year follow-up if study records indicated that they were living in England, Scotland or Wales, and had not previously withdrawn from the study or remained untraced since the previous follow-up at 53 years. Of the original cohort of 5,362 study members, 3,163 (59.0 %) were included in the target sample. Contact was not attempted with the remaining 2,198 of the original cohort who were considered ineligible for inclusion at 60–64 years: 718 (13.4 %) had died, 567 (10.6 %) lived abroad, 594 (11.1 %) were prior refusals and 320 remained permanently untraced since the last contact in 1999. Drop-outs due to death were not investigated here but previous analyses show that low childhood socioeconomic position (indicated by father being in a manual occupation, mother attaining primary level education or below, or poor housing quality) among women and low adult socioeconomic position (indicated by head of household manual occupation, not owning one’s home or low household income) among men and women predicted premature all-cause mortality (Kuh et al. 2009). Low childhood cognition, being a smoker, or having psychiatric disorder in early adulthood also predicted premature mortality (Kuh et al. 2009; Henderson et al. 2011). Of the 3,163 target sample invited to complete the postal questionnaire, between the postal questionnaire and CRF invitation, 60 died, 17 emigrated or moved out of the catchment area, and 230 were found to have an unknown address. This group of 307 were considered ineligible for inclusion in the clinical assessment mode of data collection because of the cost and participant burden of travelling, or because their whereabouts was unknown, yielding a target sample of 2,856 (Figure 1).

The primary aim was to investigate health and socio-demographic differences in response to the different elements of the study. We calculated the overall response rate by dividing the number who provided any information at the 60–64 year follow-up by the total eligible target sample. We calculated the visit cooperation rate by dividing the number who completed a CRF or home visit by the number known to be eligible for the clinical assessment (Table 2). We calculated the CRF cooperation rate by dividing the number who attended a CRF by the number who completed either a CRF or home visit.

### Explanatory measures and analysis methods

Associations between overall response rate and the visit and CRF cooperation rates and (a) socioeconomic characteristics in childhood and adulthood, and (b) adult health and health-related behaviours were examined bivariately using Wald tests. Exposures were selected to capture childhood, early adult and midlife characteristics. These were childhood cognitive ability, father’s social class in childhood, educational attainment by 26 years and housing tenure at 26 years. Cognitive ability was measured at age 8 (or at ages 11 or 15 if this was missing) using the summed score from four tests: reading comprehension, word reading, vocabulary and nonverbal reasoning (Richards et al. 2004). Father’s occupational social class at age 4 was coded according to the UK Registrar General’s Standard’s Occupation Classification. Midlife socioeconomic factors considered were economic activity, occupation-based social class (also coded to the UK Registrar General’s Standard’s Occupation Classification), housing tenure and marital status, all at 53 years. Midlife health-related factors considered were physical and cognitive performance, mental health profiles, health conditions, cardiovascular disease, obesity, smoking, physical activity and alcohol problems, at 53 years with the exception of mental health and smoking. Physical performance was evaluated utilising measures of grip strength, balance and time to rise from a chair ten times. These three indicators were summed to create an aggregate physical performance score (Guralnik et al. 2006). Cognitive performance was captured by verbal memory, measured as the number of correct words recalled from a list of 15 over three learning trials (Richards et al. 2004). Accepted thresholds indicating suboptimal cognitive and physical performance have not been defined. The 10 and 90 % cut-offs were used here in line with a previous study which aimed to evaluate functional outcomes that ‘were potentially meaningful in this middle-aged cohort’ (Guralnik et al. 2006, pp. 696). Health conditions (including cardiovascular, respiratory, cancer and other conditions) were self-reported. A further list of cardiovascular disease indicators were also self-reported (angina, leg claudication, doctor-diagnosed stroke, valvular disease, aortic stenosis). Study members were classified as obese if they had a body mass index of ≥30 kg/m^2^ based on measured height and weight. Self-reported leisure-time physical activity was assessed as the number of occasions in which study members participated in sport, vigorous leisure activities or exercises in leisure time, not including getting to and from work, in the past 4 weeks (Cooper et al. 2011). Alcohol problems were captured by the CAGE screen for potential alcohol abuse (Hatch et al. 2007). Life time smoking behaviour was derived from smoking status at 26, 31, 36, 43 and 53 years (Clennell et al. 2008). Mental health profiles based on latent classes of measures of affective signs and symptoms at 13, 15, 36, 43 and 53 years were used (Colman et al. 2007). Study members were assigned to one of four latent classes summarising their symptoms in adolescence and adulthood which can be broadly described as having symptoms in adolescence which were not present in adulthood, having adult onset symptoms, having symptoms in adolescence and adulthood and not having symptoms. Multivariable logistic regression was used to identify independent predictors of response from childhood and adult life in three steps: (i) including all socioeconomic characteristics found to be associated bivariately with response at the 20 % level of significance, (ii) including all health-related characteristics found to be bivariately associated at the 20 % level of significance and (iii) all socioeconomic and health-related characteristics identified as statistically significant predictors at the 5 % level in the two preceding steps.

Health and socioeconomic characteristics of NSHD study members were compared with those of the general population aged 60–64 years using 2001 England Census data (accessed through the Census Dissemination Unit, MIMAS (University of Manchester)). Since it was not possible to distinguish those born in and outside mainland Britain from routinely available census statistics, we additionally used data for 60–64 year olds of white ethnic origin living in England, Wales or Scotland in 2010 extracted from the Integrated Household Survey (Office for National Statistics 2010). Available sample size varies by item but is more than 28,000 for all characteristics tabled.

## Results

In total, 2,661 (84.1 %) of the target sample of 3,163 either had a visit or completed a paper questionnaire; this is the ‘overall response rate’ used for the analyses. Of the 2,661 study members who provided any data, 31 died after taking part in the postal questionnaire and before being invited to the CRF and were considered ineligible for the CRF target sample. The denominator for the ‘visit cooperation rate’ was, therefore, 2,630. The visit cooperation rate was 84.8 % (2231/2630) and the ‘CRF cooperation rate’ was 1690/2231 (75.7 %).

Of the 2,661 successfully contacted, 79 % provided information at all five of the main adult sweeps (at ages 26, 36, 43, 53 and 60–64) and 90 % gave information at four sweeps, thus providing longitudinal biological functioning data over 30 years of adulthood for the majority.

### Response at 60–64 years by childhood and adult socioeconomic characteristics

In bivariate analyses, childhood social class, educational attainment and housing tenure at 26 years were associated with overall response rate and with visit and CRF cooperation rates (Table 1). All three rates were lower among those who at 53 years were in a manual social class occupation, were not working because of long-term sickness or disability, or did not own their homes. Overall response rates and visit cooperation rates, but not CRF cooperation rates, were lower among those not married.

**Table 1:** Overall, visit and clinical research facility (CRF) response rates at 60–64 years by childhood and adult socioeconomic characteristics

	Overall response^a^ (2661/3163)	Provided clinical information at home or clinic^b^ (2,229/2,630)	Attended CRF^c^ (1690/2,229)	Mutually adjusted^d^ overall response (*n* = 2,188)	Mutually adjusted^d^ visit cooperation (*n* = 1,950)	Mutually adjusted^d^ CRF cooperation (*n* = 1,726)
	*n* (% responded)	*n* (% cooperated)	*n* (% cooperated)	OR (95 % CI)	OR (95 % CI)	OR (95 % CI)
Sex	*p* = 0.002	*p* = 0.5	*p* = 0.7	*p* = 0.002	*p* = 0.5	*p* = 0.7
Male	1,286 (82.1)	1,067 (84.3)	813 (76.2)	1	1	1
Female	1,375 (86.1)	1,162 (85.2)	877 (75.5)	1.63 (1.20, 2.23)	1.09 (0.82, 1.47)	1.05 (0.82, 1.34)
*Childhood and young adulthood*						
Childhood social class	*p* < 0.001	*p* < 0.001	*p* < 0.001	*p* = 0.7	*p* = 0.8	*p* = 0.4
Manual	1,390 (81.1)	1,134 (82.3)	790 (69.7)	1	1	1
Non-manual	1,137 (88.9)	983 (87.7)	811 (82.5)	0.94 (0.67, 1.32)	0.96 (0.70, 1.32)	0.88 (0.67, 1.15)
Educational qualifications by 26 years	*p* < 0.001	*p* < 0.001	*p* < 0.001	*p* < 0.001	*p* = 0.02	*p* < 0.001
A level & above	947 (91.2)	837 (89.4)	732 (87.5)	1	1	1
Up to GCE	705 (87.0)	604 (86.5)	468 (77.5)	0.78 (0.50, 1.21)	0.71 (0.48, 1.05)	0.56 (0.40, 0.79)
None	858 (76.2)	671 (79.2)	404 (60.2)	0.37 (0.24, 0.56)	0.56 (0.37, 0.84)	0.30 (0.21, 0.41)
Owner occupier at 26 years	*p* < 0.001	*p* = 0.001	*p* = 0.01	*p* = 0.002	*p* = 0.20	*p* = 0.9
Owner occupier	1,212 (89.5)	1,051 (88.0)	821 (78.1)	1	1	1
Non owner occupier	1,169 (82.0)	964 (83.3)	706 (73.2)	0.60 (0.44, 0.83)	0.81 (0.60, 1.09)	0.99 (0.77, 1.26)
*Adulthood*						
Social class at 53 years	*p* < 0.001	*p* < 0.001	*p* < 0.001	p > 0.9	*p* = 0.02	*p* < 0.001
Non-manual	1,622 (91.0)	1,443 (90.0)	1,201 (83.2)	1	1	1
Manual	709 (84.9)	584 (83.4)	357(61.1)	1.00 (0.71, 1.41)	0.67 (0.48, 0.93)	0.52 (0.40, 0.68)
Economic activity status at 53 years	*p* < 0.001	*p* = 0.005	*p* < 0.001	*p* = 0.04	*p* = 0.4	*p* = 0.08
Employed in paid work	2,005 (89.4)	1,748 (88.1)	1,365 (78.1)	1	1	1
Unemployed & looking	59 (81.9)	53 (91.4)	36 (67.9)	1.50 (0.56, 4.00)	1.54 (0.53, 4.44)	0.59 (0.29, 1.23)
Not looking for work/homemaker	155 (91.2)	125 (81.7)	91 (72.8)	2.82 (0.86, 9.25)	0.72 (0.37, 1.39)	0.85 (0.46, 1.55)
Retired from paid work	104 (86.7)	89 (87.3)	67 (75.3)	0.62 (0.32, 1.23)	0.74 (0.37, 1.49)	0.99 (0.51, 1.91)
Long-term sick/disabled to work	135 (70.7)	103 (78.6)	52 (50.5)	0.56 (0.32, 0.99))	1.80 (0.75, 4.29)	0.50 (0.29, 0.85)
Owner occupier at 53 years	*p* < 0.001	*p* = 0.002	*p* < 0.001	*p* = 0.002	*p* = 0.05	*p* = 0.05
Owner occupier	2,196 (90.1)	1,911 (88.1)	1,487	1	1	1
Rent privately	61 (73.5)	46 (75.4)	(77.8) 32 (69.6)	0.36 (0.19, 0.69)	0.36 (0.18, 0.75)	0.98 (0.41, 2.33)
Rent from social landlord	139 (70.9)	110 (80.3)	53 (48.2)	0.59 (0.36, 0.97)	1.04 (0.57, 1.87)	0.52 (0.32, 0.84)
Other	50 (79.4)	43 (86.0)	34 (79.1)	0.51 (0.24, 1.10)	1.00 (0.38, 2.63)	1.28 (0.53, 3.10)
Marital status at 53 years	*p* < 0.001	*p* = 0.006	*p* = 0.3	*p* = 0.01	*p* = 0.3	
Married	1,969 (89.6)	1,717 (88.1)	1,314 (76.5)	1	1	
Single/separated/divorced/widowed	487 (81.4)	400 (83.5)	297 (74.3)	0.64 (0.46, 0.90)	0.82 (0.58, 1.17)	

^a^Response rate is number providing any information divided by eligible sample

^b^Cooperation rate is number providing any clinical information at clinic or home visit divided by number providing any information less 31 study members who died between initial and CRF invitation

^c^Cooperation rate is number completing clinic visit divided by number providing any clinical information at clinic or home

^d^Logistic regression model includes all childhood and adulthood socioeconomic characteristics and sex (excluding marital status for CRF cooperation)

Blank cells indicate where characteristic not included in multiply adjusted model

Mutually adjusted models (final three columns of Table 1) showed educational attainment by 26 years predicted overall response and visit and CRF cooperation at 60–64 years. Response did not significantly vary by economic activity with the exception that those who were classified as long-term sick or disabled were less likely to respond at all or to visit a CRF. Manual social class occupation at 53 years was associated with lower likelihood of having a clinical assessment and lower likelihood of attending a CRF though not with overall response.

### Response at 60–64 years in relation to health-related characteristics

In bivariate analyses, response rate varied markedly by childhood cognitive score, being 90.0 % for those in the highest decile and 71.7 % for those in the lowest decile of cognitive score (Table 2). An exceptionally high response rate of 97.0 % was achieved amongst those in the highest decile of cognitive score at 53 years. Lower physical performance score, obesity, lifetime smoking and physical inactivity at 53 years were also bivariately associated with lower response rate and lower cooperation rates.

**Table 2:** Overall, visit and clinic visit response rates at 60–64 years by health-related characteristics

	Overall response^a^ (2661/3163)	Provided clinical information at home or clinic^b^ (2231/2,630)	Completed clinic visit^c^ (1690/2,229)	Mutually adjusted^d^ overall response (*n* = 2,143)	Mutually adjusted^e^ visit cooperation (*n* = 1,986)	Mutually adjusted^f^ CRF cooperation (*n* = 1,717)
	*n* (% responded)	*n* (% cooperated)	*n* (% cooperated)	OR (95 %CI)	OR (95 %CI)	OR (95 %CI)
*Childhood*						
Childhood cognitive score	*p* < 0.001	*p* = 0.03	*p* < 0.001	*p* = 0.01	*p* = 0.2	*p* = 0.001
Top 10 %	261 (90.0)	225 (88.3)	190 (84.4)	1	1	1
Middle 80 %	2,020 (85.6)	1,707 (85.4)	1,299 (76.1)	0.63 (0.33, 1.21)	1.22 (0.75, 1.99)	0.69 (0.44, 1.08)
Bottom 10 %	182 (71.7)	142 (79.3)	79 (55.6)	0.35 (0.16, 0.76)	0.80 (0.41, 1.57)	0.33 (0.18, 0.62)
*Adulthood*						
Verbal memory at 53 years	*p* < 0.001	*p* < 0.001	*p* < 0.001	*p* = 0.02	*p* = 0.01	*p* = 0.002
Top 10 %	327 (97.0)	299 (92.3)	259 (86.6)	1	1	1
Middle 80 %	1,875 (88.4)	1,631 (88.1)	1,248 (76.5)	0.36 (0.17, 0.76)	0.67 (0.40, 1.12)	0.59 (0.39, 0.89)
Bottom 10 %	208 (81.3)	160 (77.3)	89 (55.6)	0.39 (0.16, 0.91)	0.39 (0.21, 0.75)	0.37 (0.21, 0.63)
Physical performance score at 53 years	*p* < 0.001	*p* = 0.005	*p* < 0.001	p > 0.9	*p* = 0.4	*p* = 0.2
Top 10 %	236 (91.5)	215 (91.9)	177 (82.3)	1	1	1
Middle 80 %	1,866 (88.7)	1,615 (87.6)	1,238 (76.7)	0.95 (0.56, 1.61)	1.01 (0.60, 1.67)	0.94 (0.62, 1.42)
Bottom 10 %	190 (79.8)	152 (81.3)	96 (63.2)	0.90 (0.44, 1.86)	0.74 (0.38, 1.42)	0.63 (0.36, 1.12)
Functional limitations at 53 years	*p* < 0.001	*p* = 0.3	*p* < 0.001	*p* = 0.1		*p* = 0.5
No difficulties walking	2,263 (89.0)	1,953 (87.3)	1,516 (77.6)	1		1
Severe difficulties walking	140 (74.1)	115 (83.9)	65 (56.5)	0.71 (0.40, 1.28)		0.85 (0.50, 1.43)
Mental health profiles to 53 years	*p* = 0.4	*p* = 0.4	*p* = 0.9			
Absence of symptoms	1,275 (88.3)	1,085 (86.2)	826 (76.1)			
Repeat symptoms	567 (89.0)	499 (89.1)	377 (75.6)			
Adolescent onset, adult well	361 (88.5)	313 (87.2)	241 (77.0)			
Adult onset	221 (85.0)	192 (88.1)	143 (74.5)			
GHQ score at 53 years	*p* = 0.1	*p* = 0.3	*p* = 0.7	*p* = 0.9		
4 or fewer symptoms	1,960 (88.7)	1,682 (86.9)	1,275 (75.8)	1		
5 + symptoms	442 (86.3)	388 (88.6)	298 (76.8)	0.97 (0.67, 1.42)		
Health problems at 53 years	*p* < 0.001	*p* = 0.5	*p* = 0.01	*p* = 0.11		*p* = 0.6
No health problems	1,517 (89.8)	1,308 (87.1)	1,022 (78.1)	1		0.87 (0.67, 1.13)
1 health problem	713 (86.1)	621 (88.1)	457 (73.6)	0.72 (0.52, 1.00)		
2 + health problems	228 (81.4)	189 (85.1)	132 (69.8)	0.68 (0.40, 1.16)		0.90 (0.57, 1.43)
Cardiovascular at disease at 53 years	*p* = 0.001	*p* = 0.8	*p* = 0.08	*p* = 0.3		*p* = 0.6
No	2,337 (88.4)	2,015 (87.2)	1,540 (76.4)	1		1
Yes	121 (79.6)	103 (88.0)	71 (68.9)	0.73 (0.41, 1.32)		1.18 (0.65, 2.12)
Obese at 53 years	*p* = 0.04	*p* < 0.001	*p* < 0.001	*p* > 0.9	*p* < 0.001	*p* = 0.02
No	1,866 (88.8)	1,643 (88.9)	1,284 (78.2)	1	1	1
Yes (BMI ≥ 30kg/m^2^)	570 (85.8)	462 (82.4)	321 (69.5)	0.98 (0.70, 1.39)	0.57 (0.42, 0.77)	0.73 (0.55, g0.95)
Lifetime smoking to 53 years	*p* < 0.001	*p* < 0.001	*p* < 0.001	*p* < 0.001	*p* = 0.006	*p* < 0.001
Never smoker	751 (88.2)	644 (86.7)	514 (79.8)	1	1	1
Predominantly non-smoker	869 (89.2)	769 (89.4)	608 (79.1)	1.20 (0.80, 1.79)	1.24 (0.86, 1.80)	0.81 (0.60, 1.08)
Predominantly smoker	514 (84.8)	424 (84.0)	301 (71.0)	0.73 (0.48, 1.11)	0.80 (0.54, 1.18)	0.70 (0.50, 0.98)
Lifelong smoker	345 (76.0)	265 (77.5)	169 (63.8)	0.46 (0.30, 0.70)	0.61 (0.40, 0.92)	0.44 (0.31, 0.64)
Physical activity at 53 years	*p* < 0.001	*p* < 0.001	*p* < 0.001	*p* = 0.05	*p* = 0.07	*p* = 0.02
Very active	860 (91.0)	759 (89.3)	613 (80.8)	1	1	1
Moderately active	462 (92.2)	418 (91.3)	339 (81.0)	1.41 (0.87, 2.28)	1.52 (0.97, 2.40)	1.23 (0.87, 1.73)
Inactive	1,135 (84.1)	941 (83.9)	659 (70.0)	0.82 (0.58, 1.15)	0.91 (0.66, 1.26)	0.80 (0.61, 1.04)
CAGE alcohol problems at 53 years	*p* = 0.3	*p* = 0.3	*p* = 0.8			
No/1 problem	2,147 (88.7)	1,855 (87.4)	1,424 (76.8)			
2 + problems	167 (86.1)	148 (90.2)	115 (77.7)			

^a^Response rate is number providing any information divided by eligible sample

^b^Cooperation rate is number providing any clinical information at clinic or home visit divided by number providing any information less 31 study members who died between initial and CRF invitation

^c^Cooperation rate is number completing clinic visit divided by number providing any clinical information at clinic or home

^d^Logistic regression model includes cognitive score at 8 years and verbal memory, physical performance, functional limitations, GHQ score, health problems, cardiovascular disease, obesity, lifelong smoking, physical activity and sex all at 53 years

^e^Logistic regression model includes cognitive score at 8 years and verbal memory, physical performance, obesity, lifelong smoking, physical activity and sex all at 53 years

^f^Logistic regression model includes cognitive score at 8 years and verbal memory, physical performance, functional limitations, health problems, cardiovascular disease, obesity, lifelong smoking, physical activity and sex all at 53 years

Blank cells indicate where characteristic not included in multiply adjusted model

Cognition at 53 years and smoking behaviour emerged as independent predictors of all three response indicators. Higher childhood cognitive score was positively associated with likelihood of overall response and CRF cooperation. Being obese was inversely associated with likelihood of visit cooperation and CRF cooperation. A non-linear association between physical activity and response was seen with inactive study members having a somewhat lower likelihood of overall response and CRF cooperation.

### Multiply adjusted socioeconomic and health-related predictors of response

Independent childhood and adult social and health predictors were assessed in a final set of models (Table 3). Male gender, lower educational attainment, renting accommodation at 26 or 53 years, not being married, lower childhood cognitive score and both predominantly smoking and lifelong smoking were associated with lower likelihood of overall response. Lower educational attainment, manual social class at 53, lower childhood cognitive score, obesity, predominantly smoking and lifelong smoking were associated with lower likelihood of CRF cooperation.

**Table 3:**
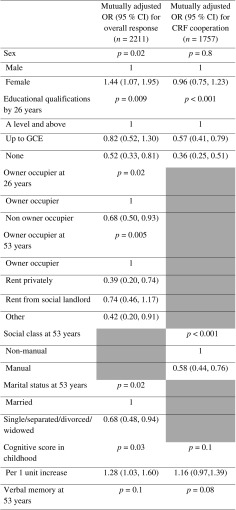

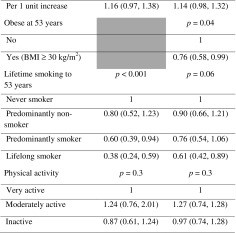
Mutually adjusted logistic regression model with socioeconomic and health-related predictors of response

Shaded cells indicate where characteristic not included in multiply adjusted model

### Comparison with general population

Table 4 compares key demographic, socioeconomic and health characteristics of 60–64 year NSHD participants with two reference populations. The sex and social class profiles were similar in the NSHD, 2001 England Census and Integrated Household Survey (IHS). Similarly small proportions were unemployed in the three data sources although a higher proportion of NSHD participants were working. The owner occupancy rate was highest in NSHD. The limiting illness rate was lower in NSHD but the smoking rate was similar to that in IHS participants.

**Table 4:** Socio-demographics of NSHD study sample at age 60–64 and general population

	NSHD study members at age 60–64:^a^ %	Relevant age band from 2001 England Census population	Profile of 2001 England Census population: %	Profile of Jan–Dec 2010 ONS Integrated Household Survey participants of white ethnic origin, aged 60–64 living in England, Scotland and Wales^b c^
Gender		60–64		
Male	48.2		48.7	48.9
Female	51.8		51.3	51.1
Own social class (NSSEC)^d^		55–64		
Higher managerial	10.6		9.6	12.2
Lower managerial	25.0		22.7	25.4
Intermediate	13.9		11.4	11.7
Small employers	14.1		13.8	12.3
Lower supervisory	8.0		9.8	9.8
Semi-routine	16.4		17.5	16.3
Routine	11.9		15.2	12.2
Employment status^e^		55–64		
Working	49.1		38.9	44.5
ILO unemployed	1.6		1.6	1.9
Retired/inactive	49.3		59.5	53.6
Housing tenure		60–64		
Owner occupier	88.8		79.4	81.6
Rent privately	3.5		5.6	5.0
Rent from social landlord	7.7		15.1	13.4
Marital status		60–64		
Married/cohabiting	77.4		72.1	71.8
Single/separated/divorced/widowed	22.6		27.9	28.2
Limiting long-term illness	26.0	60–64	33.8	not available
No limiting long-term illness	74.0		66.2	
Smoking status				
Current smoker	19.3			18.2
Ex-smoker	49.2			45.3
Never smoker	31.5			36.5

^a^Weighted for initial survey design

^b^Weighted for survey design and non-response (Integrated Household Survey User Guide—Volume 1: IHS Background & Methodology 2010)

^c^IHS data are based on core modules included in General Lifestyle Survey, Living Cost and Food Survey, English Housing Survey (EHS), Labour Force/Annual Population Survey & the Life Opportunities Survey

^d^NSSEC based on current or last main job for those who could be classified (i.e. excludes ‘never worked’ and ‘unclassified’ codes)

^e^NSHD defines working as currently in paid work. Census and HIS define working as having been in paid job in last week

## Discussion

The study achieved a high response rate at age 60–64, with 84 % of eligible study members providing some information. In line with the experience of other longitudinal studies of ageing (Menard 2007), factors likely to contribute to this high response rate include providing the option of a home visit, at least annual contact with the cohort coinciding with their birthdays and sample members sense of belonging developed through a lifetime of being part of the cohort (Pearson 2011).

Our results indicate that cognitive, rather than physical, performance was the key health-related driver of response, including visit and CRF cooperation. Childhood cognitive scores predict educational level and associated factors, probably including greater health literacy, greater social and civic engagement in a wide variety of arenas, and the confidence to take part in the various tasks comprising the latest data collection. Cognition may also reflect ability to understand and self-complete questionnaires. These findings confirm previous studies which have identified the importance of cognition for response and refusal (Deeg et al. 2002; Nishiwaki et al. 2005; Vega et al. 2010) and recent reviews which identified cognitive impairment as the key predictor of attrition in longitudinal studies (Chatfield et al. 2005; Matthews FE, Chatfield M, Brayne C: Medical Research Council Cognitive Function and Ageing Study 2006). They further suggest that cognition in childhood may be even more closely related to on-going participation than cognition in adulthood.

Psychological distress at the prior sweep and mental health profiles across several previous sweeps were not associated with response. This is similar to some previous findings (de Graaf et al. 2000) but contrasts with others which found low mood and psychiatric disease were associated with non-participation (Bootsma-van der Wiel et al. 2002; Goldberg et al. 2006).

Independently of other socioeconomic and health characteristics, manual social class and obesity predicted lower likelihood of cooperation at the clinical assessment (either at the CRF or at home), but not overall response. Obese individuals may find clinic environments more intimidating than others if they are embarrassed by their excess weight. Impaired physical function did not appear to underlie these differences.

Several other socioeconomic characteristics were investigated in relation to response patterns. Lower educational attainment was associated with lower likelihood of overall response and cooperation at the clinical assessment which, as discussed above, may reflect a range of health literacy and other factors. Lower likelihood of overall response among those not owning their accommodation may reflect lost contact due to residential mobility. However, the independent adverse effect on the likelihood of overall response of not being a home owner at 26 years in 1972 (affecting 54 % of this cohort) may also reflect lowered lifetime wealth given the young age at which it was measured and the rapidly rising British house prices since the 1970s. Previous investigation in this cohort indicates differential attrition by socioeconomic factors though has not established whether socioeconomic factors operated independently from physical health and cognition (Wadsworth et al. 1992; 2003) and a systematic review similarly did not identify socioeconomic factors as being important independent correlates of attrition, though the findings of that review were based on studies of people aged 65 and over (Chatfield et al. 2005).

### Limitations

It is challenging to identify a suitable reference population against which to compare socioeconomic, health and mortality profiles, for several reasons. First, the initial sample selection resulted in the exclusion of multiple births and those born to unmarried mothers. These groups comprised a very small proportion of the population in 1946 but a larger proportion in subsequent birth cohorts. People born outside the UK are not included in the NSHD. According to the 2001 UK Census, 90.4 % of 60–64 year olds living in England and 96.9 % in Wales classified themselves as White British. In Scotland, 99.1 % of people aged 60–64 classified themselves as White. The major in-flows of ethnic minority people occurred after 1946 and so these figures indicate that relatively small proportions of 60–64 year olds currently residing in the UK were not UK born. Second, non-response affects all studies, including the census, and so a gold standard cannot be identified. One advantage of the NSHD is that some socioeconomic, developmental and health information is available for every study member and this will facilitate the understanding and modelling of missing data.

### Implications

Cognition was strongly related to response. These results highlight the importance of designing and delivering accessible instruments. The information obtained through interviews may, for some types of data, be of higher quality than that obtained by postal surveys (Cartwright 1988; Addington-Hall et al. 1998; Tipping et al. 2010) and although interactions between educational attainment or cognitive ability and administration mode do not appear to have been investigated, we may speculate that self-completion will increasingly lead to bias as the cohort ages and cognitive deficits increase. Other approaches to data collection will need to be considered. For example, proxy responders and linked administrative data have been successfully employed to reduce bias arising from cognition-related non-response (Weir et al. 2011).

Considerable effort was made to encourage study members to attend a CRF rather than have a home visit. However, the study would have missed clinical information from 30 % of obese people, 27 % of lifelong smokers and 39 % of those from households headed by someone in a manual occupation if the option of a home visit had not been provided. Therefore, the option of a home visit is vital to collect data relevant to understanding the biology of ageing from study members with the least favourable socioeconomic and disease risk profiles (Pierce et al. 2012). The importance of including a home-based option to maximise power and reduce bias is clear and has been noted elsewhere (Kearney et al. 2011). As has been demonstrated, the key ageing traits can be captured using equipment suitable for use in the home (Guralnik et al. 2006; Stewart et al. 2001; Richards et al. 2004; Simonsick et al. 1997).

### Conclusion

The most recent NSHD sweep included physical and cognitive performance batteries, measures of the structure and function of musculoskeletal and cardiovascular body systems, and ageing biomarkers. It achieved an overall response rate of over 84 % and covered more than 2,600 men and women with longitudinal data spanning 65 years. The occupational social class and unemployment profile of on-going NSHD participants appears to be similar to the 2001 England Census reference population, though may be somewhat more advantaged with respect to home ownership and limiting illness. Whilst loss to follow-up through death has been selective, this is expected to reflect mortality patterns in the population. One advantage of the birth cohort design is that at least some data on the characteristics of those that leave the study are available. This is a unique data resource which is being used to examine child and adult developmental, social and behavioural determinants of ageing, to estimate the burden of disease and to identify risk factors that mitigate that burden among the nation’s baby boomers. Further data collection is planned to enable continued study of the lifetime factors which contribute to variations in health outcomes and their changes into advanced old age.
